# Human Rhinovirus Group C Infection in Children with Lower Respiratory Tract Infection

**DOI:** 10.3201/eid1410.080545

**Published:** 2008-10

**Authors:** Zichun Xiang, Richard Gonzalez, Zhengde Xie, Yan Xiao, Lan Chen, Yongjun Li, Chunyan Liu, Yinghui Hu, Yuan Yao, Suyun Qian, Rong Geng, Guy Vernet, Gláucia Paranhos-Baccalà, Kunling Shen, Qi Jin, Jianwei Wang

**Affiliations:** State Key Laboratory for Molecular Virology and Genetic Engineering, Beijing, People’s Republic of China (Z. Xiang, Q. Jin, J. Wang); Institute of Pathogen Biology, Beijing (Z. Xiang, R. Gonzalez, Y. Xiao, L. Chen, Q. Jin, J. Wang); Capital University of Medical Sciences, Beijing (Z. Xie, C. Liu, Y. Hu, Y. Yao, S. Qian, R. Geng, K. Shen); Fondation Mérieux, Lyon, France (R. Gonzalez, Y. Li, G. Vernet, G. Paranhos-Baccalà)

**Keywords:** HRV-C, rhinovirus, infection, children, lower respiratory tract, letter

**To the Editor:** Human rhinoviruses (HRVs), members of the family *Picornaviridae*, were first isolated in 1956 (*1*); to date, >100 serotypes have been identified on the basis of nucleotide sequence homologies. HRVs were previously divided into 2 genetic groups, HRV-A (n = 75) and HRV-B (n = 25). Recently, a putative new and distinct rhinovirus group, HRV-C, has been reportedly found in some patients with respiratory tract infections (RTIs) (*2*–*8*). To extend these initial findings and assess the pathogenicity of HRV-C, we investigated its prevalence as well as its clinical and molecular features in children with lower acute RTIs in Beijing, People’s Republic of China.

From July through December 2007, nasopharyngeal aspirates were collected from 258 children (167 boys and 91 girls) who had lower acute RTIs at the time of their admission to Beijing Children’s Hospital. The children were 1 month to 15 years of age (mean age 37 months, median age 10 months). Nucleic acids were extracted from clinical samples by using the NucliSens easyMAG platform (bioMérieux SA, Marcy L’Etoile, France). Each specimen was tested for the presence of common respiratory viruses: human parainfluenza viruses 1–4, influenza viruses, respiratory syncytial virus, enteroviruses, human coronaviruses (229E, NL63, HKU1, and OC43), metapneumovirus, adenoviruses, and bocaviruses. To study the prevalence of HRV-C, we designed a specific reverse transcription–PCR (RT-PCR) that generated a 330-bp PCR product encompassing a portion of the 5′-untranslated region, the full virus capsid protein (VP) 4 gene, and a portion of the VP2 gene of the HRV-C genome. (All primer sequences and protocols of these assays are available from J.W. upon request.)

This RT-PCR detected HRV-C in 14 patients (12 boys and 2 girls, 1 month to 13 years of age [mean age 19 months, median age 6 months]). In 6 of the 14 patients, HRV-C was the only virus detected, which suggests a direct correlation between HRV-C infection and lower acute RTIs. In the remaining 8 patients, other respiratory viruses were also detected. Respiratory syncytial virus, the most important cause of lower acute RTIs in children, was codetected in 7 of the HRV-C–positive patients, and human parainfluenza virus 3 was codetected in the other patient. Human coronavirus NL63 was codetected with respiratory syncytial virus in 1 HRV-C–positive patient.

HRV-C infection may be seasonal. This virus was detected during only 3 of the 6 months in which specimens were collected. Specifically, HRV-C was detected in samples collected in October (7/50), November (5/96), and December (2/8) but not in those collected in July (0/37), August (0/42), or September (0/25). In contrast, HRV-A and HRV-B were detected in each month (data not shown). Indeed, from July through December, HRV-A and HRV-B viruses were detected in 34 and 12 patients, respectively. Notably, in October 2007, HRV-C was detected in 7 patients, while HRV-A and HRV–B were detected in 5 and 2 patients, respectively, which suggests that the cluster of cases of HRV infections during this month was caused mainly by HRV-C.

The 14 HRV-C–positive patients had a variety of other diseases including pneumonia (6/14), bronchopneumonia (4/14), and peribronchiolitis (3/14). The most common clinical findings were cough (14/14), fever (9/14), and abnormal breath sounds on auscultation (11/14). Radiographic results were available for 10 of the 14 HRV-C–positive patients, all of whom had increased lung markings or patchy shadows. Although 3 of the 14 patients required admission to the pediatric intensive care ward, their clinical outcomes were favorable.

Phylogenetic analysis showed that the 14 sequences obtained during this study (GenBank accession nos. EU687515–EU687528) together with previously reported sequences formed a novel group of rhinoviruses (Figure). Six sequences (BCH221, BCH264, BCH200, BCH341, BCH217, and BCH250) displayed high similarity to HRV C025 (EF582386) from Hong Kong (*4*); 2 sequences (BCH249 and BCH343) displayed high similarity to NAT083 (EF077264) from the United States (*3*) and *Picornaviridae* strain 06-646 (EU081811) from Germany (*5*). BCH242 was similar to *Picornaviridae* strain tu403 (EU081795) from Germany (*5*)*.* The other 5 strains were homologous to strains from Australia (EU155152–EU155154, EU155158) (*2*,*7*). These findings suggest that, as in other countries (*5*), the HRV-C strains circulating in China are diverse. Although HRV-C strains belonging to different gene clusters cocirculate, some genetically close strains dominated during certain periods, e.g., the C025-like strain (6/14) was dominant during the study period. A similar distribution pattern is observed in epidemics of HRV-A and HRV-B (*9*,*10*).

**Figure:**
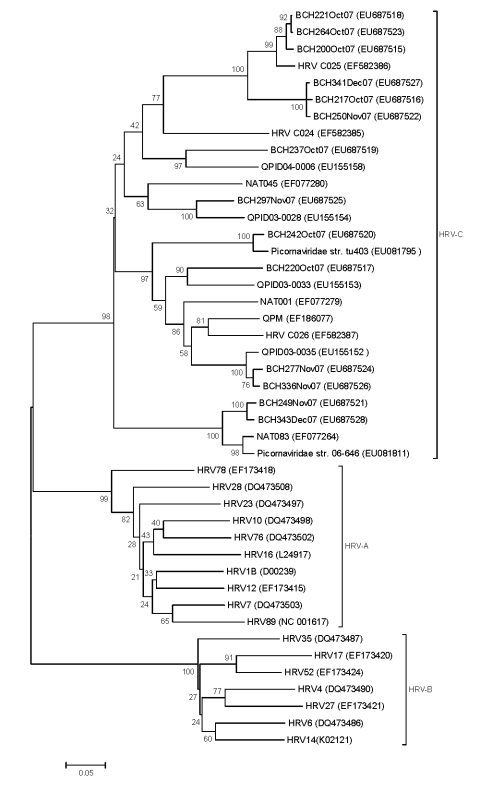
Phylogenetic analysis of the viruses detected in this study based on the nucleotide sequences of the virus capsid protein (VP)4/VP2 region. Using the VP4/VP2 nucleotide sequence (258 nt), we performed neighbor-joining analysis by applying the Kimura 2-parameter model in MEGA software version 4.0 (www.megasoftware.net). Bootstrap values from 1,000 replicates are shown next to the branches. The scale bar indicates evolutionary distance. Representative viruses from the different human respiratory virus (HRV) groups are included. GenBank accession numbers for reference sequences are indicated in parentheses.

In conclusion, HRV-C strains were detected in hospitalized children with lower acute RTIs in Beijing. Co-infections were common and complex, which indicates that the role of HRV-C in patients with multiple infections should be further investigated. Our findings provide additional evidence that HRV-C is spreading globally (*8*) and suggest that HRV-C infections should be considered a serious public health concern.
